# Beyond objective metrics: A comparative analysis of health care systems incorporating subjective dimensions to improve comparability of access and equity in healthcare assessments

**DOI:** 10.1371/journal.pone.0304834

**Published:** 2024-06-21

**Authors:** Sandra Jaworeck

**Affiliations:** Institute for Sociology, Chemnitz University of Technology, Chemnitz, Saxony, Germany; Universidad Autonoma de Chihuahua, MEXICO

## Abstract

Comparing health care systems is important for several reasons. E.g. lower-resource health care systems can learn from higher-resource ones, and country-specific progress can be made. Previous rankings of health care systems have been based on objective factors such as the number of available hospital beds or health care spending. An index is considered here that includes a subjective level that is intended to represent access to the health care system. Therefore, this study investigates the divergence between subjective and objective indices related to health care expenditure, with a focus on the influence of involuntary and voluntary payments. Utilizing the Rational Choice Theory as a framework, it explores how individual preferences and perceived benefits affect these indices. The analysis reveals that social insurance contributions, which are mandatory and beyond individual control, are evaluated differently in subjective indices compared to objective indices. This discrepancy is less pronounced for voluntary expenditures, where individuals have decision-making power. The findings highlight significant variations in the correlations between macroeconomic health care indicators and the indices, emphasizing the critical role of autonomy in financial decisions related to health care.

## Introduction

The main purpose of health care systems is to improve health with limited resources [1, 2]. Ranking health care systems accordingly is important for several reasons. First of all, to be able to compare them with each other. Comparisons strengthen better health care systems and expose weaker ones. Based on this it is easier for policymakers to make decisions and to evaluate the Sustainable Development Goals of the World Health Organization (WHO) [3]. Which is probably why the WHO made the first proposal decades ago to rank health care systems [4]. Since then, there have been several new attempts, which also differ minimally in the indicators, but mainly in the way, these indicators are weighted in the end [5].

However, all these approaches miss one crucial aspect: the access to the health care system, which is based on the subjective perception. So far, the indicators used for health care system rankings are all objective numbers, for instance: How many hospital beds are available or how much health care expenditure the country has. The question of whether people have the feeling that it is easy to get appointments and that they are being treated well has–to my best knowledge—so far been left out on the macro level.

South Africa, for example, is the country with the greatest difference between rich and poor. The health care system, especially of the private sector, there is good, but poorer people hardly have the opportunity to see a doctor locally. This means that there is a good health care system in place, but not everyone can use it in the same way [6]. This is also well known from the United States of America.

A comparison of health care systems is not only relevant for policymakers, but is also indispensable in research, especially in international comparative health research. Since cross-national data sets with surveys on objective health measures would be very expensive, the subjective level of health is used instead. In other words, country records usually contain a question about the self-assessed health, which is mostly called self-rated health.

Although self-rated health often consists of only one item, it activates complex cognitive processes at different levels, which can all be read in detail in [7]. It is important that every aspect is included of how the person rates and thinks about their own health. If you have had trouble getting an important doctor’s appointment several times and it is always been more than six months away, then this likely affects the self-rated health, especially when asked about current health. As a practical matter, studies show that it does not make much difference whether one uses subjective health or objective measurements [8].

When comparing subjective health internationally on the micro level, a health care system comparison is currently used that consists solely of objective measures on the macro level, but does not include the subjective level (e.g., access to the health care system), which can lead to incorrect conclusions being drawn from the macro to the micro level [9].

Therefore, I created a new index, which includes a subjective level on the macro level. Different health indices usually result from different weighting. The weighting chosen here is generated by a subjective attitude on the micro level. The used database contains roughly 50 different macro indicators of the Global Health Expenditure Database–GHED [10], and weighted by subjective importance [11]. For this purpose, the macro indicators were merged to the ‘International Social Survey Programme–ISSP 2011 [12]: Health and Health Care’ dataset and examined how relevant each indicator is to the link between self-rated health and confidence in the health care system. Studies show that trust in the health care system reflects the access to it [13, 14]. Thus, confidence in the health care system is the dependent variable in the index construction.

**Fig 1:**
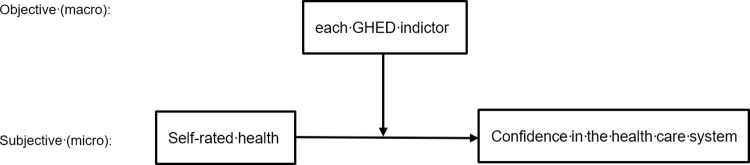
Link used for indicators weight. The arrow of GHED on the effect between self-rated health and trust in one’s own health care system does not represent a moderation effect but the weighting based on the variable importance in a causal forest.

The ISSP consists of 55.081 people from 32 countries. The survey takes place annually and has changing focus topics. So far, 2011 is the only survey wave with a focus on health and health care. In 2021, there was a new wave of ISSP focused on health. Unfortunately, the full version it is not publicly available yet [15]. Taiwan had to be excluded because the GHED dataset did not have any data for the specific period. Fig 1 shows how the individual relevance of each GHED indicator was tracked.

Accordingly, the macro indicators were weighted in an additive index and thus created a ranking including a subjective level on the macro level. The 50 indicators used cover almost all health care expenditures that exist, e.g., financing arrangements, prepayments, out-of-pocket costs, government and contributory financing schemes for health care, private (compulsory) insurance, government transfers, social insurance contributions, health insurance systems, and so forth. All indicators can be found in Table 3. In a comparison of health care systems, only indices that included health care expenditures were compared. Although only health care expenditures are available, a strong influence of the subjective level is evident. The next paragraph briefly explains how this can work.

In the end, all health care expenditures can be divided into three categories: (1) expenses that have to be made individually, (2) expenses that the government (more or less) takes over for you, and (3) expenses that you make voluntarily. After all, the only difference between the objective index and the subjective index is the weighting of the indicators. The differences should therefore occur in the correlations of macro indicators, which also have a direct impact on people or can even be influenced by people, like social insurance contributions, which belong to the first group of the three mentioned expenditures above. People must pay the expenses depending on their salary and have no influence on changing the rates, even if they are dissatisfied with it [16]. It is assumed that the indices will differ in this aspect. The evaluation of these indicators on the subjective index may differ from the ones on the objective index, because the people do not have any influence on them.

A causal forest was used to determine the variable importance of each GHED variable, see Fig 1. An additive index was formed from all GHED variables and weighted according to variable importance. After rescaling, the index takes values from zero to one hundred. The weighting represents the individual relevance of each health care expenditure and the perceived access to the health care system. Accordingly, the subjective index weights health care expenditures lower when the subjective perception—access to the healthcare system is presumed here—is worse and weights other expenditures upwards when the access to the health care system is better. In this way, the subjective level is placed on top of the objective health care expenditure, thus linking the two levels. The result is an index which has been shown to be robust and reduces country differences when comparing several multilevel models [11].

The ISSP 2011: Health and Health Care dataset was established between 2011 and 2013 [12]. From the current perspective, the used dataset is ten years old. However, more recent survey data is not available for the index construction, because, on the one hand, many countries would be required, at least 30 in order to be able to make predictions on the macro level [17], and, on the other hand, the questions about self-rated health and confidence in the health care system are necessary to build an index with a subjective level. Among the freely available data, the choice was therefore unfortunately limited.

The aim of this paper is to validate an health care index that includes a subjective level for the assessment of health care systems in different countries. The validation is intended to support the use of this index in order to be able to use self-rated health as an outcome in health research when comparing countries without creating a false conclusion from the macro to the micro level.

It is very challenging to validate an index that does not exist in this form yet. Therefore, it is not possible to show that the new index is particularly good as this question cannot be answered satisfactorily with purely statistical calculations. Rather, it will be demonstrated where differences and similarities are, and support this argumentatively. In doing so, the following questions will be addressed: How does the created subjective index differ from the same index without weighting (objective index)? When do high or low correlations show up? And from a theoretical point of view: Why do differences occur and what could this mean? Accordingly, the index is not only examined to statistical logic, but also validated in terms of content.

Hereafter, it is explained how exactly the subjective index is validated in terms of content and hypotheses proposed accordingly. Under Results, the country ranking is presented through mean differences of the countries resulting from the subjective index and compare it with the objective index as well as the Human Development Index health component. Various health-related multilevel models are calculated to compare within and between variances of these indices and determine the best estimates. Then, the correlations of the individual macro indicators with the subjective as well as the objective index will be shown. Existing and non-existing correlations between macro indicators and the subjective index as well as their possible effects regarding the subjective level are explained. Lastly, the results will be summarized, compared and discussed.

## Methods

### Validity for new achievements

There are several types of validity [18]. In this case it is primarily a matter of investigating where similarities with other health care indices lie (convergent validity) and where differences emerge (discriminant validity).

There is no statistical test which can tell how valid a new index is. Usually, one tries to build up everything that is included argumentatively, meaning one explains the operationalization and tries to present it as plausibly as possible. This is only possible to a limited extent. All available indicators were included. Thus, no preselection took place. This is due to the fact that a special method–Causal Forest [19]–was chosen for the index construction, which can handle huge amounts of data. In addition, a possible preselection should not distort the results. From a theoretical point of view, it is not necessary to make a preselection of indicators as well, since all of them have an impact on the health care system and thus on how people are doing in their country of residency.

The GHED indicators used for index construction are a total of 50 items, with only 31 countries examined, which makes a validity check difficult, since the assumption that more cases than items must exist is violated [20]. Therefore, methods such as principal component or factor analyses are questionable, and correlations are relied upon. Simultaneously, there is a limited empirical diversity, meaning that there are no more than 31 countries with this data basis.

Validity refers to a correspondence of the content of an empirical measurement with a logical measurement concept. In general, this is the degree of accuracy with which the characteristic under inspection is actually measured [21]. Thus, the new index with the subjective level should not be exactly like already existing indices, which means the ranking should vary. There should be differences that can be explained by the subjective level, but also similarities that testify to the fact that the index can meaningfully compare health care systems with each other.

#### Comparisons of various indices

For this purpose, the weighted index (with a subjective level) is considered and compared with the unweighted index (without a subjective level) and the health components of the Human Development Index (HDI). The health component measures the chance of a long and healthy life, as measured by life expectancy at birth [22]. The objective index follows the same construction as the subjective index, except that there is no weighting. Which means low index values just imply that countries invest little in their health care system, while high values suggest that countries invest a lot in their health care systems, regardless of who the investments actually stem from (individual people or their country of residency).

Resulting from this the question is: How can it be proven what distinguishes the subjective index from others? It will be based on convergent and discriminant validity. That means, first the subjective index without weights (further called ‘objective index’) is reproduced and compared with both indices (subjective and objective) by their index values and country rankings. Afterwards, both indices are compared in a multilevel regression with a health subcomponent of an already existing one, called Human Development Index [22] and look at the variance within and between countries. Lastly, correlations between the macro indicators and the two constructed indices are analyzed. Since only metric characteristics of the indices are given, Bravais-Pearson correlations are used and interpreted.

### Hypotheses

Since the subjective index contains another level, it is assumed that the subjective index can explain more than the objective index. The subjective index gives additional weight to objective health care expenditures (another level). That is why the unexplained variance in the multilevel model should be lower for the subjective index than for the other indices, which leads to Hypothesis 1.

**Hypothesis 1:** The additional level in the subjective index reduces the unexplained variance compared to the objective indices.

#### Correlations between individual GHED indicators and indices

To analyze the described phenomenon, the sociological theory of Rational Choice Theory could be helpful. This theory assumes that individuals make decisions by weighing different courses of action and choosing the one that maximizes their utility. It could explain why subjective and objective indices differ, as individual preferences and perceived utility play a significant role. People evaluate involuntary expenses (such as social insurance contributions) differently from voluntary expenses because they have no control over involuntary expenditures.

It is assumed that the subjective index has similarities with the objective index, but also relevant differences that reflect precisely the subjective level. After all, the only difference between the objective index and the subjective index is the weighting of the indicators. The differences should therefore occur in the correlations of macro indicators, which also have a direct impact on people or can even be influenced by them, like social insurance contributions, which belong to the first group of the three above mentioned expenditures. People have to pay this depending on their salary and have no influence to change the rates, even if they are dissatisfied with it [16]. In this respect, it is assumed that the indices will differ here. People will evaluate these indicators differently in the subjective index than in the objective index, because they cannot change them and are forced to pay them. Hypothesis 2 examines this link, whereby the difference between the indices is not of interest, but whether there are differences.

**Hypothesis 2:** The correlations between the GHED macro indicators and the subjective as well as objective index differ when health care expenses are imposed on people.

It is more difficult with the second group of the three spending options. Although services are covered by the state, a person still does not have the opportunity to choose from an individual benefits catalog and is accordingly forced to use the existing one. For example, the national health care system in Great Britain, is financed by taxes but is controlled by gatekeepers [23]. In Germany, the benefits can be adjusted voluntarily by means of additional services [24]. Since the choice is not completely unrestricted, it is assumed that there are also differences in the correlations between subjective and objective indices and the indicators.

Conversely, voluntary payments (third group of the three expenditures), which each person can make on their own, should not vary, because people are not forced to pay for services that they might not want. Since it is their own decision, it should not make any difference whether the service is desired or not and accordingly have the same effect on both indices, which leads to Hypothesis 3.

**Hypothesis 3:** There are barely any differences between the correlations of the GHED macro indicators and the subjective as well as objective index, when people can decide for themselves what health care expenses they will incur.

## Results

### Comparisons of various indices

#### Bivariate country comparison of objective and subjective index

**Fig 2:**
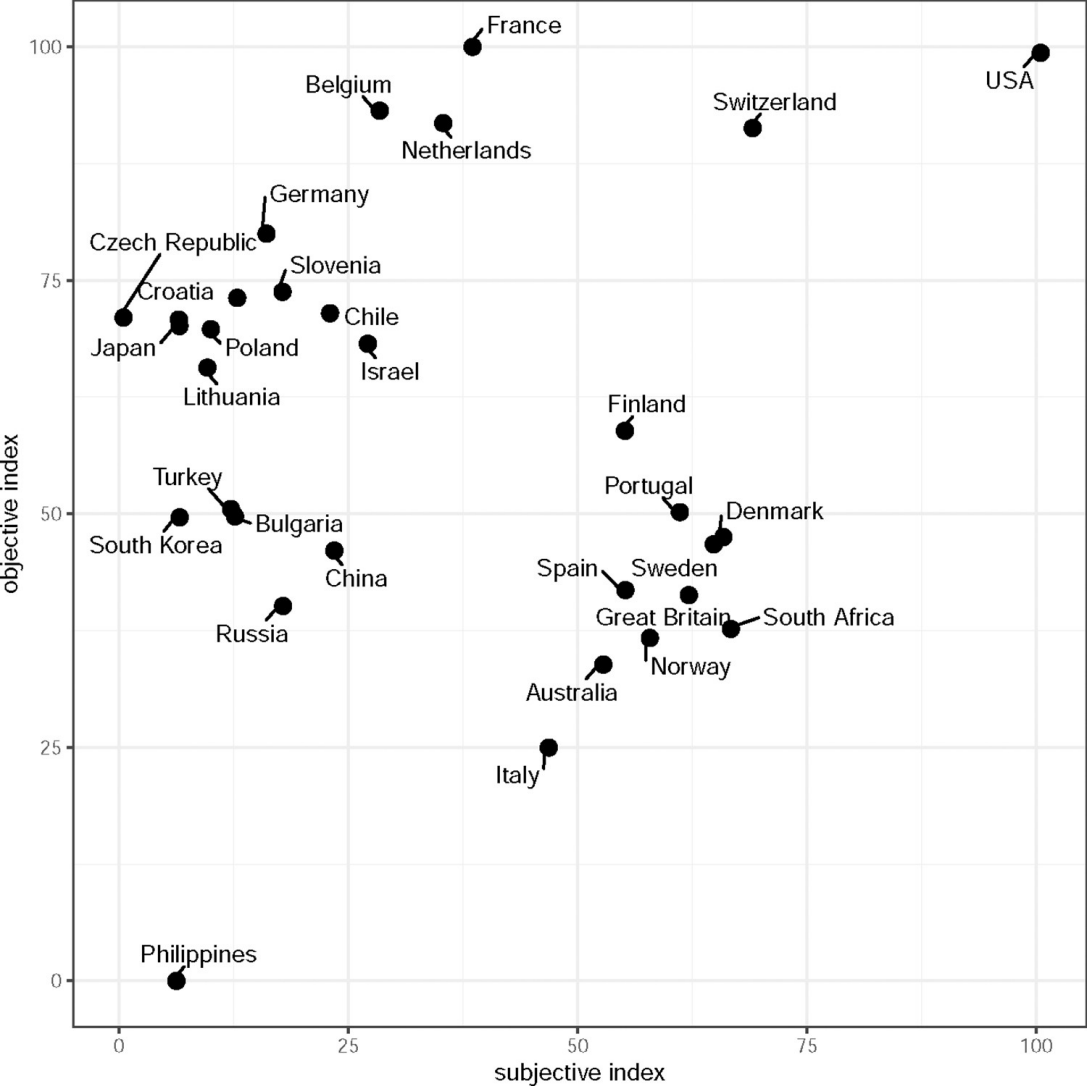
ISSP 2011. Country comparison of indices with and without subjective level (objective). USA = United States of America, N = 31.

First, the indices are compared in terms of countries and their individual index ranking as well as associated mean index values. Fig 2 shows the differences in country ranking between the subjective and the objective index. The indices agree more or less on the ranking of five countries: Philippines, Spain, Portugal, Finland, and the Middle Atlantic (USA).

The objective index rates Belgium, France, the Netherlands, Switzerland and the Middle Atlantic (USA) as the wealthiest countries. It is interesting to note that the subjective index places Belgium, the Netherlands and France in the lower midfield and the Middle Atlantic (USA) at the top, followed with a larger gap by Switzerland and South Africa.

The objective index ranks Germany, Czech Republic, Slovenia, Croatia, Japan, Poland, Chile, Israel and Lithuania in the upper midfield. The subjective index places Switzerland, Finland, Portugal, Denmark, Sweden, Spain, Great Britain, South Africa, Norway, Australia and Italy in the middle to upper midfield. Admittedly, the ranking of the subjective index seems more trustworthy. Apart from South Africa, these are all countries with a high level of prosperity in contrast to Slovenia, Czech Republic, Poland, Croatia or Lithuania according to another health care index called NUMBEO in 2012 [25].

Turkey, Bulgaria, South Korea, China and Russia are in the midfield of the objective index and have a large gap to the Philippines. In the subjective index, the Philippines are also in last place and these countries are similarly poorly positioned as Germany, Czech Republic, Slovenia, Croatia, Japan, Poland, Chile, Israel, Lithuania, Belgium, the Netherlands and France.

Even Fig 2 shows the distribution at a glance, a table is more suitable for viewing and comparing individual index values. In Table 1 the individual ranks and the corresponding index values are compared with each other.

**Table 1:** Comparison of subjective and objective index values.

Rank	Subjective index	Country	Objective index	Country
1	100	Middle Atlantic (USA)	100	France
2	68.60	Switzerland	99.35	Middle Atlantic (USA)
3	66.21	South Africa	93.16	Belgium
4	65.41	Denmark	91.84	Netherlands
5	64.35	Sweden	91.30	Switzerland
6	61.64	Great Britain	80.00	Germany
7	60.66	Portugal	73.76	Slovak Republic
8	57.38	Norway	73.13	Slovenia
9	54.71	Spain	71.48	Chile
10	54.66	Finland	71.01	Czech Republic
11	52.30	Australia	70.81	Croatia
12	46.36	Italy	70.11	Japan
13	38.05	France	69.77	Poland
14	34.86	Netherlands	68.20	Israel
15	27.93	Belgium	65.65	Lithuania
16	26.64	Israel	58.89	Finland
17	22.98	China	50.53	South Korea
18	22.51	Chile	50.19	Portugal
19	17.40	Russia	49.72	Turkey
20	17.33	Slovenia	49.63	Bulgaria
21	15.58	Germany	47.51	Denmark
22	12.39	Slovak Republic	46.75	Sweden
23	12.14	Bulgaria	46.07	China
24	11.70	Turkey	41.84	Spain
25	9.52	Poland	41.30	Great Britain
26	9.14	Lithuania	40.14	Russia
27	6.122	South Korea	37.69	Norway
28	6.08	Japan	36.72	South Africa
29	6.03	Croatia	33.87	Australia
30	5.76	Philippines	24.98	Italy
31	0	Czech Republic	0	Philippines
Mean overall	34.80		60.50	

* Own calculations. Database: ISSP 2011 and GHED.

Although both indices are built on the same scale, the weighting of the subjective index has a strong influence on the individual index values. The mean value of the subjective index is only about half (34.8) in contrast to the objective index (60.5). However, there are also countries that have a higher index value after weighting, for example Denmark with 65.41 in contrast to the objective index value of 47.51. The same applies to Sweden, Great Britain, Portugal, Norway, Spain, Australia, Italy, South Africa and the Philippines, with South Africa making the biggest skip. Whereby the Philippines are only minimally improved upwards (from 0 to 5.76). All other countries are downgraded by the subjective weighting further than in the objective index value. It can be seen that the objective index has a spread of 24.98% between the two lowest countries (Philippines and Italy). The subjective index does not have this spread. On the other hand, the subjective index has a margin of 31.4% between the top two countries (Middle Atlantic (USA) and Switzerland). This shows the influence of the subjective level for all countries, except for the Middle Atlantic (USA).

In the objective index, for example, France and the Middle Atlantic (USA) are on an equal footing. This means that they spend roughly the same amount of money according to the data overall. If the weighting for the subjective index is now built in and the two countries are compared, the Middle Atlantic (USA) lands in first place, while France drops down to rank 13. Although France takes first place with an objective index value of 100, it only has a value of 38.05 for the subjective index.

In the United States of America, the individual freedom is of great importance. On average, people from the United States of America tend to rate themselves better in health than the rest of the world [own calculation using ISSP 2011]. Likewise, it is questionable how many poorly positioned people are included in the survey that they cannot afford their own housing and thus are not registered. These would most likely give different answers than financially well-positioned people who could also help themselves in the greatest need [26]. These characteristics probably lead to the result that the Middle Atlantic (USA) ranked first. Simultaneously, this could also be one of the reasons why South Africa comes off so well in the ranking.

#### Hypothesis 1

The health-related multilevel model is estimated with the dependent variable of self-rated health and includes the following control variables: physical activities, education, age, and sex.

Physical activity does not only prevent physical problems. It can also be integrated into the treatment and rehabilitation of various diseases. People who are physically active on average consider themselves to be healthier and have fewer health problems [27]. Sports behavior is surveyed with the question: How often do you exercise physically for at least 20 min so that you sweat or breathe more than usual? No sport at all is the reference, while the other outcomes are: once a month or less often, several times a month, several times a week and daily.

The proportion of people with mental impairments is smaller among those with higher educational status [28], implying that educational background is positively related to health outcomes [29]. Primary school is the reference, while the other five outcomes are: lower secondary, upper secondary, post-secondary, non-tertiary, lower level tertiary, first stage and upper level tertiary.

Longitudinal studies show that self-rated health changes with age. Physical health objectively deteriorates with age, but people tend to think that they are healthier than they are as they get older [30]. Age was queried openly and limited in the analysis to people between 18 and 82 years of age in order to define a uniform upper and lower limit (18 years of the actual possible characteristics) to reduce possible survival effects (distortions in favor of survivors) [31].

Men and women differ in their self-rated health and illness [32]. Based on the survey results, it is also important to consider the gender health paradox, which describes the observation that men consider themselves to be healthier than women, even though the latter have a higher life expectancy [33]. Sex was asked dichotomously by asking if one is a woman or a man. No alternative information could be given.

There is no multicollinearity in the models (each VIF value is smaller than 4.7). The estimator of the subjective index is statistically significant at the 5% level (p = 0.031). The objective index as well as the HDI health components are not statistically significant. Since the individual estimators of the covariates are not of interest here, only the variances of the models and the estimators of the indices are presented, see Table 2.

**Table 2:** Variance comparison of the models.

	Subjective index	Objective index	HDI health components
*σ* ^2^	513.97	513.97	513.97
*τ*_00 country_	30.88	35.29	35.22
ICC	0.06	0.06	0.06
Marginal R^2^	0.128	0.119	0.120
Conditional R^2^	0.177	0.175	0.176
Estimator	[0.01,0.16]	[-0.06,0.13]	[-0.22,0.49]
N _Individual_	45.836		
N _Countries_	31		

* Own calculations. Database: ISSP 2011 and GHED.

The estimators of the variables in the different models behave as assumed and differ only minimally, if at all: self-rated health increases the more one does exercise (p < 0.007), the more educated (p < 0.001), the younger one is (p < 0.001), and if one is a man (p < 0.001).

The within group variance is not explained by the model (*σ*^2^) is for the subjective as well as the objective index and the health components of the HDI the same. Which makes sense, since the countries do not change within. In addition, the following finding supports the subjective index: *τ*_*00*_ country decreases from the objective as well as the HDI health components to the subjective index. This suggests that the deviation of countries from overall average decreases more for the subjective index. The ICC is the same for all indices, which means that there are the same differences between countries. The Conditional as well as the marginal R^2^ is bigger at the subjective index in comparison to the others and it is the only model with a significant positive subjective index estimator (p = 0.031). Hypothesis 1 can therefore be supported. The subjective index reduces the unexplained variance and leads to better model parameters than the objective indices.

The next step is to look at the individual correlations between the subjective index and the objective index in order to filter out similarities and differences on a theoretical basis. These are then used to explain what exactly distinguishes the subjective and objective indices from each other and thus to find out what the subjective level actually represents.

### Correlations between individual GHED indicators and indices

In order to determine the underlying cause of the differences, Table 3 shows the correlations of the indices with each GHED indicator used. In addition, the absolute difference between the two indices is given and the weights of each indicator (weight), which was used to build the subjective index. This shows which indicators were weighted particularly high or low.

**Table 3:** Comparison of subjective and objective index values.

	GHED indicator	r_objective_	r_subjective_	difference	weight
1	current health expenditure (CHE) as % of gross domestic product (GDP)	0.180	0.087	0.093	0.015
2	health capital expenditure as % of GDP	0.162	0.127	0.035	0.018
3	domestic health expenditure as % of CHE	0.207	-0.090	0.297	0.006
4	domestic general government health expenditure as % of CHE	0.006	0.081	0.075	0.008
5	domestic private health expenditure as % of CHE	0.011	-0.090	0.101	0.017
6	voluntary health insurance as % of CHE	-0.052	-0.042	0.010	0.013
7	out-of-pocket as % of CHE	-0.198	-0.099	0.099	0.012
8	other private health expenditure as % of CHE	0.222	-0.016	0.238	0.029
9	external health expenditure as % of CHE	-0.199	0.067	0.266	0.009
10	compulsory financing arrangements as % of CHE	0.214	0.079	0.135	0.006
11	government financing arrangements as % of CHE	-0.430	0.288	0.718	0.043
12	compulsory health insurance as % of CHE	0.520	-0.249	0.769	0.011
13	social health insurance (SHI) as % of CHE	0.381	-0.363	0.744	0.005
14	voluntary financing arrangements as % of CHE	-0.215	-0.078	0.137	0.009
15	rest of the world as % of CHE	0.095	-0.195	0.290	0.022
16	compulsory private health insurance as % of CHE	0.399	0.289	0.110	0.011
17	government subsidy to social health insurance as % of SHI	0.184	0.015	0.169	0.014
18	self-employed contributions to social health insurance as % of SHI	-0.229	-0.021	0.208	0.006
19	general government expenditure as % of GDP	0.164	0.042	0.122	0.017
*health expenditure data (all in % of GDP)–revenues*					
20	current health expenditure by revenues of health care financing schemes	0.037	0.042	0.005	0.021
21	transfers from government domestic revenue (allocated to health purposes)-	-0.373	0.306	0.679	0.034
22	international transfer and grants international transfer and grants	-0.383	0.299	0.682	0.012
23	transfers by government on behalf of specific groups	0.187	-0.210	0.397	0.007
24	subsidies	0.125	0.340	0.215	0.007
25	other transfers from government domestic revenue	-0.250	0.187	0.437	0.007
26	transfers distributed by government from foreign origin	0.083	-0.160	0.243	0.068
27	social insurance contribution	0.435	-0.296	0.731	0.002
28	social insurance contribution from employees	0.409	-0.412	0.821	0.009
29	social insurance contribution from employers	0.242	-0.355	0.597	0.006
30	social insurance contribution from self-employed	0.055	-0.296	0.351	0.009
31	other social insurance contributions	0.294	-0.186	0.480	0.010
32	compulsory prepayment	0.329	0.028	0.301	0.023
33	voluntary prepayment	-0.044	-0.071	0.027	0.115
34	other domestic revenues	-0.009	-0.132	0.123	0.020
35	other revenues from households	-0.059	-0.097	0.038	0.006
36	other revenues from corporations	0.200	0.127	0.073	0.031
37	other revenues from non-profit institutions serving households (NPISH)	-0.136	-0.214	0.078	0.044
38	direct foreign transfers	-0.151	0.117	0.268	0.006
39	current health expenditure by financing schemes	0.037	0.042	0.005	0.025
40	government schemes and compulsory contributory health care financing schemes	0.085	0.083	0.002	0.028
41	schemes	-0.415	0.322	0.737	0.009
42	compulsory contributory health insurance schemes	0.518	-0.288	0.806	0.004
43	social health insurance schemes	0.386	-0.443	0.829	0.003
44	compulsory private insurance scheme	0.388	0.281	0.107	0.001
45	voluntary health care payment schemes	-0.056	-0.013	0.043	0.064
46	voluntary health insurance schemes	-0.079	-0.035	0.044	0.095
47	non-profit institutions serving households financing scheme	-0.195	-0.165	0.030	0.008
48	enterprise financing scheme	-0.088	0.351	0.439	0.017
49	household out-of-pocket payment	-0.057	-0.101	0.044	0.006
50	rest of the world financing schemes (non-resident)	0.082	-0.059	0.141	0.004
51	gross domestic product in US dollar per capita	0.091	0.107	0.016	
52	objective index (without subjective weights)	1	0.001	0.999	

* r = Bravais-Pearson correlation, objective = index without weights, subjective = index with weights, difference = absolute difference between correlation of the subjective and the objective index, weights = weights for the subjective index.

Often, the correlations at least headed in the same direction. However, there are indicators where deviations occur and the sign of the correlation changes accordingly, which is the case for (11) government financing arrangements, (12) compulsory health insurance, (13) social health insurance, (21) transfers from government domestic revenue, (22) international transfer and grants, (23) transfers by government on behalf of specific groups, (25) other transfers from government domestic revenue, (27–31) all social insurance contributions, (41) government schemes, (42) compulsory contributory health insurance schemes, (43) social health insurance schemes and (48) enterprise financing scheme.

The individual correlations and the difference between them are interesting, but it makes it easier to get an overview if this table is converted into percentages and summarized. A distinction is made between five categories. Indicators that are more or less the same between the subjective and objective index show a difference of less than 15% percent. Indicators that show a slight difference are between five and 15%, with a medium influence between 15% and 25%, with a big effect between 25% and 35% and with most impact over 35%, see Table 4.

**Table 4:** Proportion of influence on the diversity of indices.

[0% - 5%]	Current health expenditure; health capital expenditure, domestic general government health expenditure, voluntary health insurance, out-of-pocket, current health expenditure by revenues of health care financing schemes, voluntary prepayment. other revenues from households, other revenues from corporations, other revenues from non-profit institutions serving households, current health expenditure by financing schemes, government and compulsory contributory health care financing schemes, voluntary health care payment schemes, voluntary health insurance schemes, non-profit institutions serving household financing scheme, rest of the world financing schemes
[5% - 15%]	domestic health expenditure, domestic private health expenditure, other private health expenditure, external health expenditure, compulsory financing arrangements, voluntary financing arrangements, rest of the world, compulsory private health insurance, government subsidy to social health insurance, self-employed contributions to social health insurance, general government expenditure, subsidies, transfers distributed by government from foreign origin, compulsory prepayment, other domestic revenues, direct foreign transfers, compulsory private insurance scheme, household out-of-pocket payment
[15% - 25%]	transfers by government on behalf of specific groups, other transfers from government domestic revenue, social insurance contribution from employees, enterprise financing scheme
[25% - 35%]	transfers from government domestic revenue, international transfer and grants, social insurance contribution from employers, other social insurance contributions
[35% +]	government financing arrangements, compulsory health insurance, social health insurance, social insurance contribution, social insurance contribution from employees, government schemes, compulsory contributory health insurance schemes, social health insurance schemes

All the before and above mentioned indicators distinguish the subjective from the objective index. These indicators each account for a difference between the indices of at least 15%. Each social insurance contribution has a larger impact on the subjective and the objective index. While those correlate positively with the objective index, they show a negative correlation with the subjective index, which supports Hypothesis 2. For the objective index it seems obvious that the more contributions, the more payments for the health care system are available. In contrast, it has a negative effect on the subjective index if people do not have the possibility to determine for themselves, for example, their social insurance contributions.

While government financing schemes are negatively correlated with the objective index, they are positively correlated with compulsory health insurance and social health insurance. For the subjective index, this is the other way around, which also supports Hypothesis 2. Government financing arrangements do not directly impact people, but health insurance does.

In the case of (other) transfers from the government’s domestic revenues and internal transfers and grants, a negative correlation with the objective index and positive correlations with the subjective index are shown. This is to be interpreted analogously to the government financing schemes, which does not explain why it is the other way around for transfers by the government on behalf of specific groups.

Even if there is occasionally a change of sign in other correlations between the GHED macro indicators and the indices, the correlations do not differ enough that it seems worth mentioning (less than 15%).

Differences in correlations are below five percent for the following variables: (1) current health expenditure, (2) health capital expenditure, (4) general domestic health expenditures, (6) voluntary health insurance, (7) out-of-pocket costs, (20) current health expenditure by revenues of health care financing schemes, (33) voluntary prepayment, (35) other revenues from households, (36) other revenues from corporations, (37) other revenues from non-profit institutions serving households, (39) current health expenditure by financing schemes, (40) government and compulsory contributory health care financing schemes, (45) voluntary health care payment schemes, (46) voluntary health insurance schemes, (47) non-profit institutions serving household financing scheme and (50) rest of the world financing schemes (non-resident).

A large proportion of these are indicators that are ‘mandated’ but do not directly affect one (current health expenditures, health investments, other revenues). With the exception of voluntary financing arrangements, all indicators for voluntary payment are also in the first group. Voluntary financing arrangements have only a very small effect and, at 0.133, just exceeded the 0.1 limit, see Table 3, which supports Hypothesis 3. When people can make their own decisions regarding health care expenses, their perceptions of access to the health care system, as reflected in the indices, remain stable.

If in addition the second category is considered in which differences up to 15% are imposed, then the correlations between expenditures on health care are very similar. This means that they have the same effect on both indices, which is slightly positive and these also do not directly affect the human being.

Interestingly four of those which make almost no difference to the indices have a very large weighting in the creation of the subjective index. These include (33) voluntary prepayment, (37) other revenues from non-profit institutions serving households, (45) voluntary health care payment schemes and (46) voluntary health insurance schemes, see Table 4.

This shows that despite high weighting within the subjective index, the differences to the objective index are not only in the strong weighting factors, but totality is responsible for a variation. Transfers distributed by the government from foreign origin are also weighted quite high in the subjective index, making up a difference of less than 15 percent. The only indicator that is highly weighted in the subjective index, but in the sixth rank, is the government financing arrangements, which differ very much between the correlations of the subjective and objective index (more than 35%). The results shown here are discussed in the next section.

## Discussion

It is likely that there are several latent levels behind the subjective index. One is the macro level from the GHED data set for the countries. This was combined with the ISSP 2011 country data set consisting of over 50.000 individuals who provided information on their self-rated health and confidence in their health care system. This approach was chosen to consider the subjective level at the macro level in case self-rated health is the desired outcome variable in further studies. The subjective index will be used to evaluate residents’ access to their health care system.

For this purpose, the country rankings were first considered: With the exception of South Africa and the Middle Atlantic, the assignment of countries by the subjective index seems plausible. In South Africa, extreme poverty and extreme wealth coexist. Inequality from the apartheid era persists after more than 20 years of democracy [6]. This could be a decisive reason for South Africa achieving such a high ranking. The private health care system in South Africa is well developed meanwhile. People who are included in the sample may be wealthier, which is why they are more likely to be in the private health care sector. It is also a pity that the United States of America ranked first due to its very high health care spending. This is often a problem with health care system indices. It is well known that the United States of America does not have the best access to the health care system, because it is socio-economically conditioned. The ranking nevertheless seems reasonable, since people who self-insure also know to what extent they are insured and what access they have to certain services. What might additionally put the United States of America at the top is the excellence in medical education. People from all over the world who can afford it travel to the United States of America to receive the best treatment from specialists. The arrangement of the remaining countries does not seem unlikely. The fact that the subjective index also differs from NUMBEO is a good indication that other levels are considered. Since it is not exactly known what is involved in the subjective level, it could in addition to access to the health care system also be assumed that the number of hours of sunshine or the influence of the latitude within a country plays a role [34, 35].

In the multivariate model, the subjective index already showed robustness [11]. Now the subjective index was compared with the health component of the Human Development Index and with the same index without subjective weights. Although the same estimators and statistical significances were found for the control variables across all models, only the subjective index was statistically significant (p = 0.031). At the same time, the model with the subjective index has the highest explained variance (R^2^_subjective_ = 12.8, R^2^_objective_ = 11.9, R^2^_HDI_ = 12.0) and the deviation of countries from the overall average decreases more for the subjective index (*τ*_00 country_subjective_ = 30.88, *τ*_00 country_objective_ = 35.29, *τ*_00 country_HDI_ = 35.22).

It is now necessary to discuss whether these statements also appear plausible in terms of content. Correlations between expenditures on health care are very similar overall indices. This means that they have the same effect on both indices. This makes sense, since in this respect expenditure on health cannot be influenced by an individual. Neither can compulsory financing arrangements, prepayments, private insurances, transfers from abroad distributed by the government, and so forth. It is different from voluntary payments or financing arrangements. It must not be forgotten that even if they are voluntary, the schemes are already in place and the individual only has the option to choose a product or not, but cannot look for one with individual benefits. According to the applied Rational Choice Theory this could be the reason why even voluntary benefits do not show any difference between the indices.

Social insurance contributions have a different impact on the subjective and the objective index. While these correlate positively with the objective index (the more contributions, the more payments for the health care system), they show a negative correlation with the subjective index. This means that it is no longer possible to assume a pure measurement of payments and further levels are identified in the subjective index. The more social security contributions, the worse the subjective index turns out to be. People who pay a lot of social security contributions often sit in higher positions and have to bear more responsibility, which can also result in distress [36]. It is quite possible that precisely these people do not feel well taken care of in their health care system due to the increased risk of falling ill.

Government financing arrangements are rated positively in the subjective index. This could be because it is perceived as positive that these financing arrangements exist at all. In contrast, it has a negative effect on compulsory contributory health insurance and social health insurance. This affects the individual directly and one cannot choose one’s corresponding benefit. The state financing arrangements affect one only indirectly and one is grateful to be able to fall back on them in various situations.

That (other) transfers from the government’s domestic revenues and internal transfers and grants are positively associated with the subjective index show that people perceive them as helpful in accessing the health care system. After all, these come from the state and have a supportive effect. This is contradicted by the fact that the situation is the other way around when it comes to transfers from the state for specific groups. Perhaps because this is about specific groups where people are nevertheless directly affected.

Without question, this paper is not without limitations. On the one hand, as mentioned at the beginning, it is difficult to validate something that has not existed in this form before. This also includes the data selection, which left little room for choice. Data had to be taken that were exclusively available for the financing of health care systems, since there were no other freely available data in the 31-country comparison. Now, this train of thought, on the other hand, is difficult to link to a subjective level, but a suggestion has been made about how a subjective level could also play a role in the form of health care spending. It is claimed that the index represents access to the health care system. Even though the paper provides good content reasons to assume this different countries also have different cultural and political characteristics, which are included in the equation. The very distinction between National Health Systems and Social Security Systems has a major impact, too. It is possible that the index also reflects these, but it cannot be said exactly.

Despite these limitations, the subjective index also offers some significant advantages. It allows for the consideration of aspects of access to the healthcare system that may not be captured by purely objective indicators. This could contribute to a more comprehensive understanding of how people perceive and utilize their health care systems.

Furthermore, the subjective index provides a means to capture the significance of factors such as government financing arrangements and social security contributions for the perception of the health care system. This can provide important insights into how governmental interventions and policy decisions influence the perception and access to the health care system.

Overall, it can be observed that the subjective index represents a promising tool for evaluating health care systems, providing valuable insights beyond purely objective indicators. However, future research should conduct further validation studies and utilize a more comprehensive database to enhance the reliability and relevance of the subjective index and expand its potential applications.

Future research should clarify the results presented here. For this purpose, larger country data sets would be beneficial. Likewise, it would be good to have more than just the health care expenditures for the Causal Forest and accordingly the calculation of the subjective weights.

## Conclusion

This research underscores the significant divergence between subjective and objective indices in evaluating health care expenditures, highlighting the critical role of individual autonomy in financial decision-making related to healthcare. The subjective level of the index, calculated based on confidence in the health care system through various expenditures, differs notably from the objective index. This study finds that social insurance contributions, which are mandatory and beyond individual control, correlate negatively with subjective indices and positively with objective ones. This divergence is minimal for voluntary expenditures, where individuals exercise choice, reflecting a stable perception across indices.

The subjective index not only holds statistical significance in the multilevel model but also indicates that the direct impact of health care spending on individuals versus their freedom to choose their insurance significantly affects their evaluations. According to the Rational Choice Theory this makes sense; when people can choose their health care benefits, they likely prioritize what is personally important, leading to more favorable subjective assessments.

Moreover, the correlations between GHED macro indicators and the indices vary in meaningful ways, with plausible country rankings further validating these differences. It is evident that numerous latent variables influence the perceived ’access to the healthcare system’. Despite this complexity, the subjective index can be reasonably interpreted as a measure of access to healthcare, leading to the designation of this measure as the Health Care Assessment Index. This new index provides valuable insights into how financial autonomy and compulsion in health-related expenditures impact overall perceptions, offering important implications for health policy and economic planning.
